# Heavy metal accumulation during the last 30 years in the Karnaphuli River estuary, Chittagong, Bangladesh

**DOI:** 10.1186/s40064-016-3749-1

**Published:** 2016-12-06

**Authors:** Ai-jun Wang, Ahmed Kawser, Yong-hang Xu, Xiang Ye, Seema Rani, Ke-liang Chen

**Affiliations:** 1 Third Institute of Oceanography, State Oceanic Administration, Xiamen, 361005 China; 2 Department of Oceanography, University of Dhaka, Dhaka, 1000 Bangladesh

**Keywords:** Heavy metal, Sedimentation rate, Pollution load index, Catastrophic events, Karnaphuli River estuary

## Abstract

Heavy metal contamination of aquatic environment has attracted global attention owing to its abundance, persistence, and environmental toxicity, especially in developing countries like Bangladesh. Five heavy metals, namely chromium (Cr), copper (Cu), nickel (Ni), lead (Pb) and zinc (Zn) were investigated in surface and core sediments of the Karnaphuli River (KR) estuary in Chittagong, Bangladesh, in order to reveal the heavy metal contamination history in estuarine sediments and its response to catastrophic events and human activities. The surface sediment was predominantly composed of silt and sand, and the surface sediment was contaminated with Cr and Pb. Based on the ^210^Pb chronology, the sedimentation rate in the inter-tidal zone of KR estuary was 1.02 cm/a before 2007, and 1.14 cm/a after 2008. The core sediment collected from 8 to 20 cm below the surface mainly originated from terrestrial materials induced by catastrophic events such as cyclone, heavy rainfall and landslides in 2007 and 2008. The values of contamination factor (*CF*) showed that the sediment became moderately contaminated with Cr and Pb in the last 30 years. The variation and accumulation of heavy metals in core sediment before 2000 was mainly related to natural variations in sediment sources; however, in subsequent years, the anthropogenic inputs of heavy metals have increased due to rapid physical growth of urban and industrial areas in the Chittagong city. In general, the accumulation pattern of heavy metals after normalization to Aluminum in sediments of KR estuary indicated an accelerated rate of urbanization and industrialization in the last 30 years, and also suggested the influence of natural catastrophic event on estuarine environment.

## Background

A conceptual environmental problem associated with urbanization and industrialization in space and time is the increased heavy metal pollution (Nriagu 1990). Originating from various anthropogenic and natural sources, metals are eventually released into aquatic or atmospheric systems, although the anthropogenic inputs in environments have increased dramatically since the Industrial Revolution (Nriagu 1979; Thevenon et al. 2011). Bangladesh being a developing country is experiencing rapid industrial developments and unplanned urban growth in recent years (Mia et al. 2015), but pollutants produced by these activities are triggering environmental problems on an unprecedented scale, primarily due to arsenic and other heavy metal pollution (Tareq et al. 2003; Bhuiyan et al. 2011; Islam et al. 2015a; Sharifuzzaman et al. 2016). For example, the aquatic ecosystems of Chittagong, which is a second largest city, main seaport and economic nerve-centre of the country, is under multiple stresses due to discharge of effluents from textile and cement industries, ship recycling, oil refineries, tanneries, paint manufacturing and dyeing plants, paper and rayon mills, naval and merchant ships, steel and engineering factories, fertilizer and other chemical industries as well as disposal of sewage and solid wastes directly into the adjacent Karnaphuli River (KR) and coastal waters of Bay of Bengal (Chowdhury et al. 1999; Hossain and Khan 2002; Ali et al. 2016). The presence of toxic and hazardous substances in river, estuary and marine environments not only affects the ecosystem integrity (Rahman et al. 2014) but also poses substantial threats to public health and welfare in Bangladesh (Alam et al. 2003; Islam et al. 2014).

Estuary, a special location, is formed at the mouths of rivers, in the narrow boundary zone between the sea and the land, and interactions between physical, chemical and biological processes within an estuary can have profound influences on the transport and fate of substances discharged from the river system (Dyer 1997; Bianchi 2007). Due to the special geographic and hydrodynamic characteristics, estuary serves as the main sink for most heavy metals discharged by river system (Dassenakis et al. 1995; Ip et al. 2004; Zhang et al. 2009; Xia et al. 2011; Delgado et al. 2012; Xu et al. 2014), and the estuarine sediment can provide short- or long-term record of the accumulation of trace metal inputs from riverine, atmospheric, and anthropogenic sources (Windom et al. 1988; Alexander et al. 1993; Ip et al. 2004; Delgado et al. 2012; Xu et al. 2014). Moreover, the study of heavy metal contamination in estuarine sediment does not only indicate the environment health issue (Li et al. 2007) but also reveals information on relevant human activities in the surrounding areas (Delgado et al. 2012). Although a number of studies investigated the heavy metal contamination and seasonal variations in water and sediment compartments of Karnaphuli River (KR) and within the river catchment (Islam et al. 2013, 2015b; Dey et al. 2015; Ali et al. 2016), only few studies were carried out in the estuarine environment. Therefore, the objectives of this study are to survey the contamination with heavy metals and reveal their accumulation history in the sediment of KR estuary, and further discuss the results in relation to catastrophic events and human activities.

## Methods

### Study area

**Fig. 1:**
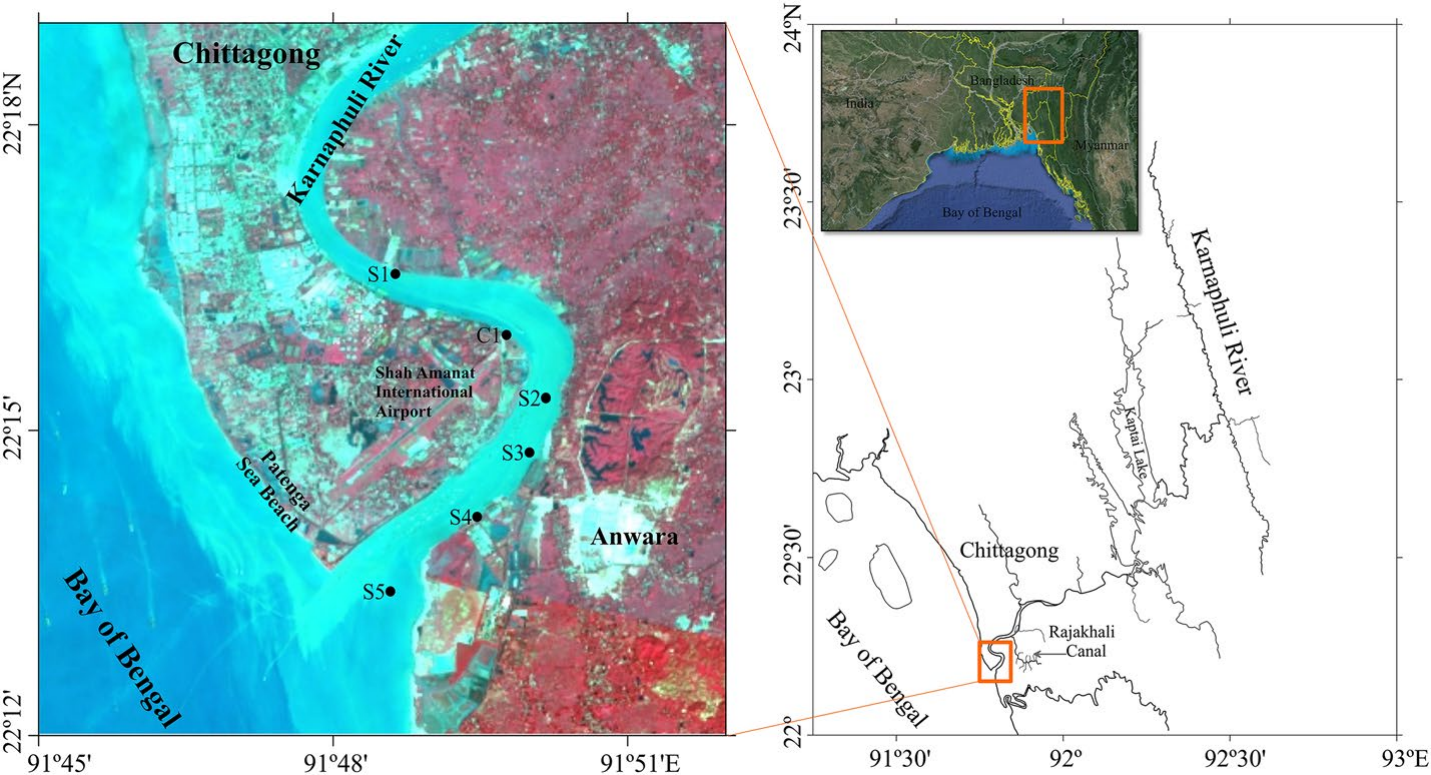
Location of the sediment sampling sites (*left*) in the study area (*right*)

The Karnaphuli River is one of the major and most important rivers in Chittagong and the Chittagong hill tracts. The river originates from the Lushai Hills of Mizoram in India with a catchment area of approximately 11,000 km^2^ (Ahmed et al. 2013), travels through 180 km of mountainous wilderness at Rangamati in Bangladesh and then flows through the port city of Chittagong about 170 km, and discharges into the Bay of Bengal (Fig. 1). People of Chittagong city are dependent on KR for drinking water and other house hold purposes. Geologically, the entire river catchment consists of a substratum of tertiary rocks covered with alluvial deposits, and the overlying deposits comprise successive layers of mud and sand (Rizbi 1971). KR estuary, which is located between latitude 22°53′N and longitude 91°47′E near Patenga in Chittagong city, is one of the most important estuaries in Bangladesh. The estuary is characterized by semidiurnal tides with 2–4 m range and an average channel depth of 8–10 m in the external zone (Lara et al. 2009). Because of profound influence of Indian monsoon, the environmental parameters in KR estuary fluctuate seasonally (Alam and Zafar 2012).

### Collection of sediment samples

Five surface sediments, S1–S5 (upper 5 cm of the river-bed surface), and a short core (C1) were collected from KR estuary in September, 2014 (autumn) (Fig. 1). The surface sediment was collected by fisher-man diving near the river bank within 1–2 m water depth, and the short core was collected from inter-tidal flat using PVC pipe pushed into the sediment. Collected surface sediment samples were put into polythene bags and sediment core sample in the PVC pipeline with PVC caps. All samples were immediately stored at 4 °C in icebox and brought to the laboratory for pretreatment and further analysis.

### Analytical methods

The core was split and sediment was sliced with at 1 cm interval after studying sedimentary structure description in the laboratory. The surface and core sediments were analyzed for grain-size, total organic carbon (TOC) and heavy metals. The core sediments were also analyzed for radioisotope of ^210^Pb to dating the sedimentation year.

Grain-size analyses were performed with a Mastersize 2000 laser particle size analyzer (Malvern, UK). The mean grain-size (Mz), sorting coefficient (σ), skewness (Sk_1_) and kurtosis (K_G_) were calculated using graphic method (Folk and Ward 1957). The sediment type was classified using the ternary textural diagram of Shepard (1954). TOC was analyzed with an *Elementar Vario EL*-*III* element analyzer (made in Germany) following the method described by Ye et al. (2014). Replicate analyses of acetanilide as standard yielded a mean precision of about 0.3% for organic carbon. Heavy metal concentrations were measured using an OPTIMA 7300DV ICP-AES following Xu et al. (2014). The relative standard deviation for each element was less than 5%, and the precision of duplicate samples was <10% (RSD).

The ^210^Pb radioactivity at different depths of sediment core was detected by α spectrometer. After addition of the ^209^Po yield tracer, 5 g of dried sediment sample was digested by nitric acid and hydrochloric acid. Subsequently, the sample was leached with 1 N hydrochloric acid, and pH of the clear solution was adjusted to two using ammonia. After heating and stirring, ^209^Po and ^210^Po were allowed to deposit onto the silver slides that were rinsed with pure water and ethanol, and then dried under an infrared lamp for the measurement. The sedimentation rate for sediment core was estimated after subtracting background value as recommended by Li et al. (2002).

### Assessment of heavy metals in sediment

Contamination factor (*CF*) and pollution load index (*PLI*) have been extensively used to assess heavy metal contamination in sediment in recent years (Islam et al. 2015a, b, c; Ali et al. 2016). The contamination factor (*CF*) of metals was estimated using Eq. (1) as below:

1 $$CF_{\text{metal}} = C_{\text{metal}} /C_{\text{background}}$$

where *C*_metal_ is the heavy metal concentration in sediment and *C*_background_ is the background value of that heavy metal. Therefore, the choice of background value of heavy metal plays significant role in assessment of heavy metal contamination. In this paper, we selected the background value of each heavy metal as recommended by Banu (1995). The *CF* was classified into four grades for assessing the pollution of one single metal over a period of time: low degree (*CF* < 1), moderate degree (1 ≤ *CF* < 3), considerable degree (3 ≤ *CF* < 6), and very high degree (*CF* ≥ 6) of pollution. The pollution load index (*PLI*) was estimated using Eq. (2) as below:

2 $$PLI = \, (CF_{1} \times CF_{2} \times \cdots \times CF_{n} )^{1/n}$$

where, *CF*_*n*_ is the contamination factor of *n*th metal. When *PLI* < 1, indicates there is no pollution; and *PLI* > 1 means the sediment is polluted (Islam et al. 2015c; Ali et al. 2016).

## Results and discussion

### Surface sediment properties

The results of surface sediment properties are summarized in Table 1. The silt and clay contents of surface sediment increased from upstream to the river mouth, while sand content showed a decreasing trend. The average percentages of silt, clay and sand in the sediment were 65.14, 12.18, and 22.69%, respectively. Typically sediment particles can be classified into two main types: sandy silt (ST) and silt (T). The mean grain size (Mz) of surface sediment varied from 17.02 to 35.70 μm. However, Mz decreased correspondingly from upstream to the river mouth (Table 1). The sorting coefficient (σ) ranged from 1.76 to 2.28, signifying that surface sediment in the estuary was poorly sorted. The skewness (Sk_1_) of surface sediment at different points ranged from −0.08 to 0.35, and in most cases positively skewed. The kurtosis (K_G_) of surface sediment ranged 0.95–1.14, indicating a mesokurtic distribution. Total organic carbon (TOC) content in sediments varied from 0.43 to 0.71%, showing a decrease from upstream to the river mouth (Table 1).

**Table 1:** Surface sediment composition, grain-size parameters and total organic carbon content

Site	Longitude	Latitude	Sand/%	Silt/%	Clay/%	Type	Mz/μm	σ	Sk_1_	K_G_	TOC/%
S1	91°48′38″E	22°16′32″N	29.78	59.44	10.78	ST	35.70	1.89	0.35	0.95	0.69
S2	91°50′10″E	22°15′19″N	30.33	58.80	10.87	ST	34.71	1.93	0.31	0.96	0.58
S3	91°50′00″E	22°14′47″N	11.91	73.94	14.15	T	17.02	1.83	0.11	1.14	0.43
S4	91°49′28″E	22°14′09″N	17.97	70.74	11.29	T	27.02	1.76	0.31	1.02	0.71
S5	91°48′35″E	22°13′25″N	23.45	62.74	13.81	ST	18.51	2.28	−0.08	1.05	0.46

### Depth-distribution of sediment properties and dating

**Fig. 2:**
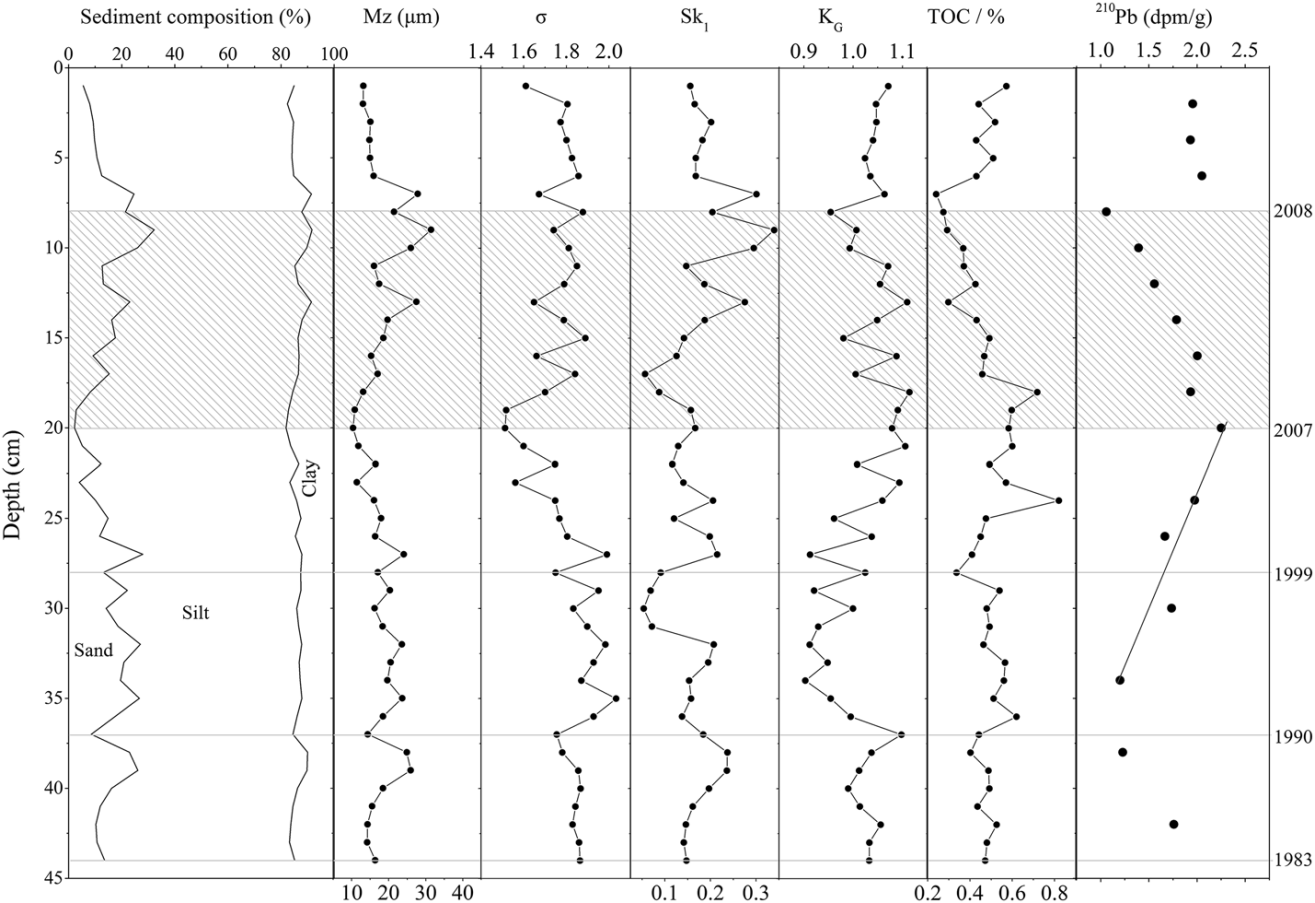
Depth-distribution of core sediment composition, grain-size parameters, TOC content and ^210^Pb radioactivity in the Karnaphuli River estuary, Bangladesh

Sediment along the vertical profile was composed of dark grey mud with no obvious laminated structure. The major component of sediment in the core was silt (71.14%) followed by sand (15.12%) and clay (13.74%). The sediment particles can be classified into three types: sandy silt (ST), silt (T) and clayey silt (YT). The mean grain-size was comparatively small (up to 15 μm) close to the surface, but found to increase (up to 35 μm) between 8 and 20 cm, and then decreased again at the bottom part. The sorting coefficient and skewness also showed the same variations along the profile. The vertical variation of sediment grain-size parameters suggested that the sampling station, in particular below 20 cm, is characterized by a stable sedimentary environment like the evolution of typical inter-tidal flat environment (Ren et al. 1983; Bartholdy and Madsen 1985). TOC contents along the profile were 0.24–0.82% with a mean value of 0.48%, and the depth-variation was not obvious except for the layer between 8 and 20 cm where levels of TOC decreased gradually (Fig. 2).

The radioactivity of ^210^Pb demonstrated a complicated variation along the profile. Based on the above mentioned facts, the variations of all core sediment properties showed an abnormal sediment layer: 8–20 cm below the surface. In this layer, the intensity of ^210^Pb radioactivity decreased upward, indicating that this sedimentary layer was inverted (Fig. 2). This inverted sedimentary structure may be induced by catastrophic events. Between 2007 and 2008, the city of Chittagong experienced cyclone “Akash”, heavy rainfall (the most pronounced rainfall event since 1982) and landslides in many locations, within 1–4 km of the KR (Lara et al. 2009; Khan et al. 2012). Thus huge terrestrial materials were washed into the river that potentially led to obvious environmental variations in the estuary (Lara et al. 2009). If we hypothesize that the sediment between 8 and 20 cm below the surface was deposited between 2007 and 2008 because of natural catastrophic events, the mean sedimentation rate since 2008 was 1.14 cm/a. Below 20 cm, the intense radioactivity decayed downward. Then the mean sedimentation rate was 1.02 cm/a using the method recommended by Li et al. (2002).

### Heavy metal concentrations in sediment

Heavy metal concentrations in surface sediments of KR estuary are presented in Table 2. Metal contents (dry weight) ranged over following intervals: Al: 4.67–5.92%, Cr: 77.70–99.08 mg/kg, Cu: 20.34–33.06 mg/kg, Ni: 34.10–41.27 mg/kg, Pb: 23.66–25.05 mg/kg, Zn: 59.69–74.32 mg/kg. Their mean concentrations were 5.45%, 87.42, 25.92, 38.26, 24.20 and 65.53 mg/kg, respectively. When compared with background value of these metals in KR (Banu 1995), concentrations of Cr and Pb exceeded the background value in estuary area, and concentrations of other three metals were less than that of background value. This data suggest that the surface sediment of KR estuary is contaminated with Cr and Pb.

**Table 2:** Concentrations of Al and other heavy metal in surface and core sediment

Site	Layer (cm)	Al (%)	Cr (mg/kg)	Cu (mg/kg)	Ni (mg/kg)	Pb (mg/kg)	Zn (mg/kg)
S1	0–5	4.67	77.70	20.34	35.01	24.26	59.69
S2	0–5	4.85	78.39	20.54	34.10	24.35	61.37
S3	0–5	5.86	99.08	32.26	41.27	23.66	74.32
S4	0–5	5.92	91.38	23.38	39.82	23.70	66.36
S5	0–5	5.92	90.54	33.06	41.10	25.05	65.89
C1	0–1	2.80	102.30	25.44	42.15	24.98	70.18
	2–3	4.47	102.70	34.86	48.03	25.44	77.97
	4–5	4.27	100.90	34.24	48.05	25.38	77.28
	6–7	3.93	79.95	17.82	33.67	23.08	50.75
	8–9	4.51	79.69	18.70	32.46	23.15	50.93
	10–11	4.92	92.19	26.24	40.89	25.54	66.52
	12–13	4.95	97.22	32.28	43.96	26.61	73.56
	14–15	4.42	104.60	34.43	47.17	25.63	82.10
	16–17	5.31	83.69	23.92	38.26	23.51	64.23
	18–19	6.26	119.70	58.17	56.20	27.46	116.30
	20–21	5.68	119.30	45.93	55.82	28.38	101.40
	22–23	5.34	111.80	42.04	51.93	27.17	88.73
	24–25	4.75	99.62	31.88	42.84	25.05	70.54
	26–27	4.78	88.27	25.76	38.22	25.47	67.72
	28–29	4.78	91.85	28.83	39.78	24.91	69.27
	30–31	4.14	88.60	26.77	38.78	24.42	64.02
	32–33	4.16	90.48	26.72	38.69	25.22	66.07
	34–35	4.97	87.21	27.04	38.43	24.09	66.32
	36–37	4.76	89.53	27.07	39.41	24.90	71.91
	38–39	4.26	90.27	30.09	39.03	25.96	64.81
	40–41	5.82	101.60	35.97	43.75	26.25	77.97
	42–43	6.45	105.30	36.39	47.08	24.68	87.96
Background value^a^			77.20	33.00	56.10	22.80	95.00

^a^Data from Banu (1995)

**Fig. 3:**
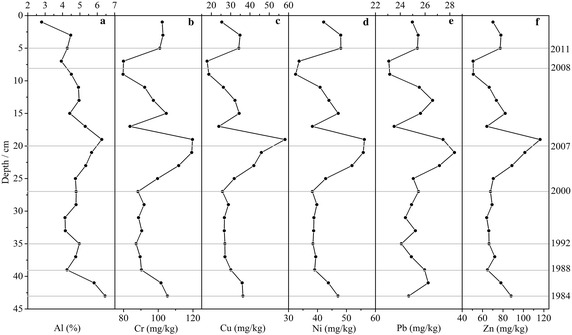
Depth-distribution of Al and other heavy metal concentrations in core sediment in Karnaphuli River estuary, Bangladesh

The concentration profiles of Al, Cr, Cu, Ni, Pb and Zn in sediment core are displayed in Fig. 3. Al content ranged from 2.80 to 6.45% with a mean value of 4.81% along the profile, but the temporal evolution of other heavy metals indicates complex variability. The concentrations of Cr, Cu, Ni, Pb and Zn ranged from 79.69 to 119.70, 17.82 to 58.17, 32.46 to 56.20, 23.08 to 28.38, and 50.75 to 117.30 mg/kg, respectively. The mean value of these metals were 96.67, 31.39, 42.94, 25.33 and 73.93 mg/kg (n = 22), respectively. The down-core profiles of these metals were characterized by similar variations identical to Al. As shown in Fig. 3, all heavy metals considered in this study decreased from 1983 to 1988, and sustained a stable variations from 1988 to 2000, and then increased sharply from 2000 to 2007. However, heavy metal concentrations decreased again from 2007 to 2008, and then increased rapidly since 2008. In comparison with background value (Banu 1995), contamination level of heavy metals was similar to those determined in surface sediments, e.g., sediments along the core were contaminated with Cr and Pb in recent 30 years, and only with Cu and Zn in around 2007. However, contamination with Ni was not noted.

The correlation matrix for analyzed sediment parameters was calculated to examine any interrelation among the parameters and the results were presented in Tables 3 and 4. The elements in surface sediments showed significant positive correlation among them except for Pb suggesting those metals were from similar sources. On the other hand, similar kind of results was also obtained for metals in core sediments where Pb showed no significant correlation with other metals.

**Table 3:** Correlation matrix showing the relationship between the trace metal and surface sediment properties (number: 5, threshold value of relation coefficient at a 95% confidence level: 0.878)

	Mz	TOC	Al	Cr	Cu	Ni	Pb	Zn
Mz	1.000							
TOC	0.772	1.000						
Al	−0.887	−0.443	1.000					
Cr	−0.924	−0.568	0.914	1.000				
Cu	−0.974	−0.854	0.782	0.819	1.000			
Ni	−0.950	−0.536	0.963	0.943	0.877	1.000		
Pb	0.008	−0.272	−0.134	−0.362	0.192	−0.100	1.000	
Zn	−0.872	−0.650	0.784	0.962	0.778	0.830	−0.451	1.000

**Table 4:** Correlation matrix showing the relationship between the trace metal and core sediment properties (number: 21, threshold value of relation coefficient at a 95% confidence level: 0.503)

	Mz	TOC	Al	Cr	Cu	Ni	Pb	Zn
Mz	1.000							
TOC	−0.701	1.000						
Al	−0.552	0.313	1.000					
Cr	−0.742	0.637	0.617	1.000				
Cu	−0.709	0.667	0.661	0.957	1.000			
Ni	−0.769	0.647	0.585	0.983	0.947	1.000		
Pb	−0.542	0.531	0.451	0.854	0.835	0.832	1.000	
Zn	−0.756	0.663	0.714	0.953	0.978	0.951	0.806	1.000

### Assessment of heavy metal pollution during the last 30 years

**Fig. 4:**
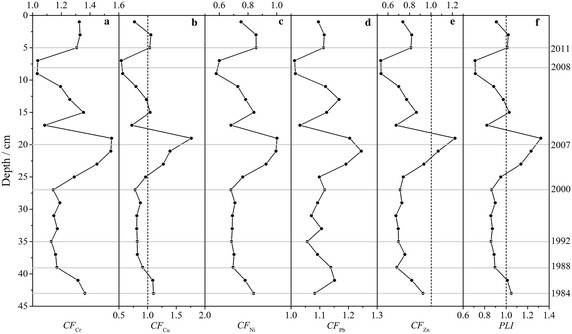
Contamination factor (*CF*) and pollution load index (*PLI*) value of heavy metals in the last 30 years in Karnaphuli River estuary, Bangladesh. (*Dot line of the vertical axis* indicates the baseline level of pollutants)

The calculated *CF* and *PLI* values of heavy metals in sediments were presented in Fig. 4 and Table 5. Before 2000, *CF* and *PLI* values of heavy metals in sediments varied uniformly, and since then increased rapid until to 2007 when *CF* and *PLI* reached highest value. However, from 2007 to 2008, *CF* and *PLI* values of heavy metals decreased rapidly which may be induced by a series of catastrophic events discussed in the next section. From 2008, *CF* and *PLI* values of heavy metals increased again. The degree of contamination with different heavy metals as indicated by *CF* was: Cr > Pb > Cu > Zn > Ni (high to low). There was no Ni contamination in sediment in the last 30 years. There was moderate degree of contamination with Cr and Pb in the last 30 years, whereas, moderate degree of contamination with Cu and Zn around 2007. The *PLI* values ranged from 0.71 to 1.32 indicating that the sediment was contaminated in some time stages, and the high *PLI* values occurred during 2004–2007 period.

**Table 5:** Contamination factor (*CF*) and Pollution load index (*PLI*) value of heavy metals in surface and core sediments

Site	Layer (cm)	*CF*_Cr_	*CF*_Cu_	*CF*_Ni_	*CF*_Pb_	*CF*_Zn_	*PLI*
S1	0–5	1.01	0.62	0.62	1.06	0.63	0.76
S2	0–5	1.02	0.62	0.61	1.07	0.65	0.77
S3	0–5	1.28	0.98	0.74	1.04	0.78	0.94
S4	0–5	1.18	0.71	0.71	1.04	0.70	0.85
S5	0–5	1.17	1.00	0.73	1.10	0.69	0.92
C1	0–1	1.33	0.77	0.75	1.10	0.74	0.91
	2–3	1.33	1.06	0.86	1.12	0.82	1.02
	4–5	1.31	1.04	0.86	1.11	0.81	1.01
	6–7	1.04	0.54	0.60	1.01	0.53	0.71
	8–9	1.03	0.57	0.58	1.02	0.54	0.71
	10–11	1.19	0.80	0.73	1.12	0.70	0.88
	12–13	1.26	0.98	0.78	1.17	0.77	0.97
	14–15	1.35	1.04	0.84	1.12	0.86	1.03
	16–17	1.08	0.72	0.68	1.03	0.68	0.82
	18–19	1.55	1.76	1.00	1.20	1.22	1.32
	20–21	1.55	1.39	1.00	1.24	1.07	1.23
	22–23	1.45	1.27	0.93	1.19	0.93	1.14
	24–25	1.29	0.97	0.76	1.10	0.74	0.95
	26–27	1.14	0.78	0.68	1.12	0.71	0.86
	28–29	1.19	0.87	0.71	1.09	0.73	0.90
	30–31	1.15	0.81	0.69	1.07	0.67	0.86
	32–33	1.17	0.81	0.69	1.11	0.70	0.87
	34–35	1.13	0.82	0.69	1.06	0.70	0.86
	36–37	1.16	0.82	0.70	1.09	0.76	0.89
	38–39	1.17	0.91	0.70	1.14	0.68	0.90
	40–41	1.32	1.09	0.78	1.15	0.82	1.01
	42–43	1.36	1.10	0.84	1.08	0.93	1.05

### Influence of human activities and catastrophic events on heavy metal accumulation

KR estuary is located in the city of Chittagong which is experiencing rapid and frequent economic development activities (Mia et al. 2015), and human activities reported to collide with nature that eventually affecting the estuarine environment and ecosystems.

**Fig. 5:**
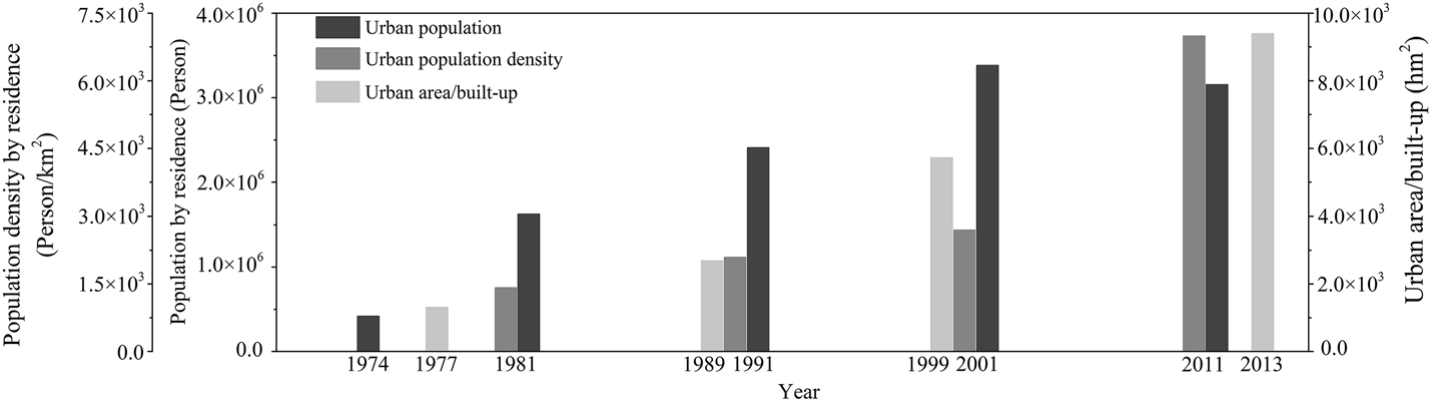
Population, population density by residence and urban area/built-up in Chittagong in the last 30 years

Since early 1980s, Chittagong experienced a rapid urbanization (Fig. 5). The urban population by residence in Chittagong increased from 0.42 million in 1974 to 3.15 million in 2011, and the urban population density by residence increased from 1413 Person/km^2^ in 1981 to 6992 Person/km^2^ in 2011 (Bangladesh Bureau of Statistics 2015), while the urban area/built-up (including commercial, residential, industrial, and other infrastructure) increased from 1309.68 hm^2^ in 1977 to 9401.85 hm^2^ in 2013 (Hassan and Nazem 2015). As the city expanded, the industries (such as spinning mills, dying, cotton, textile, steel mills, oil refineries, leather, paints, fertilizer, and others) were constructed along the river correspondingly, and these industries discharged huge amount of untreated effluents to KR which induce high concentration of heavy metals in the water and sediments, especially for Pb (Majid et al. 2003; Ali et al. 2016). Previous studies displayed that the concentrations of heavy metals in surface water of KR demonstrated little indication of metal pollution in 1997–1998 that did not reach alarming levels (Das et al. 2002; Majid et al. 2003), and the metal contamination continued slowly to 2011–2012 (Dey et al. 2015); however, since 2011, the water quality of KR has decreased considerably, mainly due to elevated levels of heavy metals that rendered the waters from this river unsuitable for direct drinking and/or cooking in 2015 (Ali et al. 2016).

**Fig. 6:**
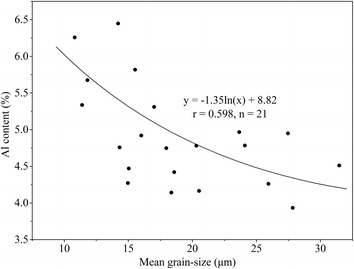
Relationship between mean grain-size and Al content in core sediment

**Fig. 7:**
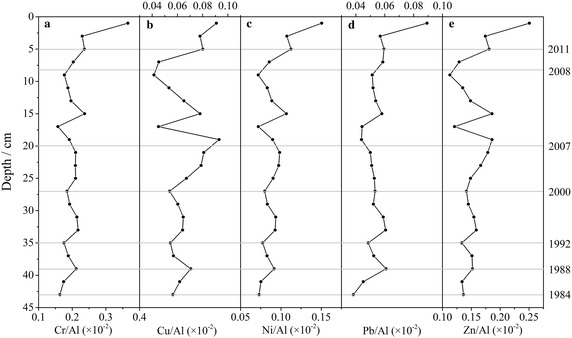
Depth-distribution of the ratios of heavy metals and Al in core sediment

In many cases, anthropogenic inputs exceed natural background levels from weathering of rock materials described earlier. Moreover, large variations in the concentrations of metals in estuarine sediments may arise due to poorly sorted mixtures of sand, silt and clay. Thus, there needs to be a way to separate background levels from anthropogenic inputs and to account for the natural variability of sediment composition. One preferred method is to normalize trace element concentrations to a carrier phase (Summers et al. 1996). In the case of elemental ratios, Al (Aluminum) is often chosen as a normalization element because of high natural abundance in crustal rocks, while its concentration in anthropogenic sources is generally low. Therefore, the ratio of ‘metal:Al’ has effectively been used as an indicator of pollution sources in river and coastal systems (Windom et al. 1988; Summers et al. 1996). Down-core profiles of normalized heavy metal concentrations have also been effectively used to examine historical profiles of contaminant inputs to estuaries (Alexander et al. 1993). There is a statistically good relationship between Al content and mean grain-size of core sediment sample at a 95% confidence level (Fig. 6), suggesting that variations of Al content was related to sediment properties and could be considered as the lithogenic effects. The depth-distribution of heavy metals was derived after normalization by Al (Fig. 7), which was very different compared to Fig. 3.

After normalization, variations of ‘metal:Al’ ratio indicate variability of anthropogenic heavy metal inputs. According to Fig. 7, the anthropogenic inputs of heavy metals increased from 1984 to 1988, and decreased from 1988 to 1992, and then inputs remained relatively input until 2000. Since then, the anthropogenic contributions of heavy metals were not synchronous. Specifically, Cu and Zn increased rapidly, and Ni showed a slight increase, although Cr and Pb were stable from 2000 to 2007. Between 2007 and 2008, due to natural catastrophic events in Chittagong (Lara et al. 2009; Khan et al. 2012), a huge amount of terrestrial sediments induced by landslides were brought into KR river by floodwater (Lara et al. 2009). Eventually those sediments with low metal content were transported to estuary and accumulated in the strata that resulted in reduced inputs of Cu, Ni and Zn, although Pb content was increasing at that stage. Pb is expected to add to soils because of not only effluents from paint industries during industrialization (Majid et al. 2003), but also emission from vehicle and living during urbanization (Kibria et al. 2013), because there is no waste water treatment plant exists for the urban water waters until now in Chittagong, and few industries do have waste water treatment plant for their industrial waster waters to be cleaned. During heavy rainfall, Pb contaminated soils are flushed into the river and through this process anthropogenic input of Pb enhanced in estuarine sediments. Since 2008, the inputs of all heavy metals from anthropogenic sources increased rapidly that caused a certain contamination of some heavy metals (Ali et al. 2016).

Therefore, the level of heavy metal accumulation, after normalization by Al, in sediments of KR estuary indicated an influence of urbanization and industrialization in recent 30 years as well as the influence of catastrophic events on estuarine environment.

## Conclusions

The pollution of aquatic ecosystems by heavy metals is emerging as a serious issue in Bangladesh. Using the ^210^Pb dating method, this study determined heavy metal accumulation recorded in a single sediment core (C1) collected from KR estuary, Chittagong. The contamination levels of Cr and Pb in surface sediment were higher than their background values, indicating that sediments of KR estuary have been polluted with Cr and Pb. Excluding the influence of natural catastrophic events, KR estuary enjoys the fairly stable sedimentary environment with constant sedimentation rate of about 1 cm/a. Since 2000, the anthropogenic metal inputs to sediments enhanced due to increase urbanization and industrialization. Catastrophic events (such as landslides, cyclones, heavy rainfall, etc.), between 2007 and 2008, also led to changes in source materials and depositional environment of estuary, and thus altered metal accumulation in sediments. In general, heavy metal enrichment in KR estuary is likely to be associated with accelerated urban and industrial growth in recent 30 years, including catastrophic events in the area.

## Abbreviations

KR: Karnaphyli River
*CF*: contamination factor
*PLI*: pollution load index
TOC: total organic carbon
